# Comparison of interactions between beta-hairpin decapeptides and SDS/DPC micelles from experimental and simulation data

**DOI:** 10.1186/1471-2091-8-11

**Published:** 2007-07-16

**Authors:** Allison A Langham, Alan J Waring, YN Kaznessis

**Affiliations:** 1 Department of Chemical Engineering and Materials Science, 421 Washington Ave SE, Minneapolis, MN 55455, USA; 2 Department of Medicine, David Geffen School of Medicine at UCLA, Los Angeles, CA 90095, USA; 3 Department of Pediatrics, David Geffen School of Medicine at UCLA, Los Angeles, CA 90095, USA; 4 Digital Technology Center, University of Minnesota, 421 Washington Ave SE, Minneapolis, MN 55455, USA

## Abstract

**Background:**

We applied a combined experimental and computational approach to ascertain how peptides interact with host and microbial membrane surrogates, in order to validate simulation methodology we hope will enable the development of insights applicable to the design of novel antimicrobial peptides. We studied the interactions of two truncated versions of the potent, but cytotoxic, antimicrobial octadecapeptide protegrin-1, PC-72 [LCYCRRRFCVC] and PC-73 [CYCRRRFCVC].

**Results:**

We used a combination of FTIR, fluorescence spectroscopy and molecular dynamics simulations to examine the peptides' interactions with sodium dodecylsulfate (SDS) and dodecylphosphocholine (DPC) micelles. The relative amounts of secondary structure determined by FTIR agreed with those from the simulations. Fluorescence spectroscopy, deuterium exchange experiments and the simulations all indicate that neither peptide embeds itself deeply into the micelle core. Although molecular simulations placed both peptides at the micelle-water interface, further examination revealed differences in how certain residues interacted with the micelle core.

**Conclusion:**

We demonstrate here the accuracy of molecular dynamics simulations methods through comparison with experiments, and have used the simulation results to enhance the understanding of how these two peptides interact with the two types of micelles. We find agreement between simulation and experimental results in the final structure of the peptides and in the peptides final conformation with respect to the micelle. Looking in depth at the peptide interactions, we find differences in the interactions between the two peptides from the simulation data; Leu-1 on PC-72 interacts strongly with the SDS micelle, though the interaction is not persistent – the residue withdraws and inserts into the micelle throughout the simulation.

## 1. Background

Antimicrobial peptides (AMPs) are produced by many, if not all, plants and animals [1-3]. Despite over two decades of study, the mechanism of action of AMPs against cellular and microbial membranes is not entirely clear, hindering efforts to design novel, non-toxic antimicrobial peptides [4]. Many AMPs target the membrane lipid bilayer, as evidenced by experiments showing that their presence increases the rate of internal leakage from synthetic liposomes. Furthermore, enantiomeric versions of many AMPs are as active as their native counterparts, suggesting that stereospecific receptors are not the targets of AMPs [5,6]. An important factor hindering development of therapeutic AMPs is that many active antimicrobial peptides also injure human cells, and thus would benefit from structural modifications that reduce host-toxicity levels without impairing their potency against pathogens.

We undertook this study believing that a combined experimental and computational approach that clarifies how peptides interact with mammalian host and microbial membranes could be a valuable adjunct to AMP-design. It may one day be possible to pinpoint sequence regions or residues that contribute to peptide activity or toxicity by examining the interactions of specific residues with membrane mimics, though the study of numerous peptides will be required to reach this goal. This study combines experimental techniques such as Fourier-transform infrared spectroscopy (FTIR) with atomistic molecular dynamics (MD) simulations to determine the validity of the simulation methodology and to demonstrate the utility of MD simulations in providing molecular level detail about the interactions between the peptides and membrane mimics. After confirming the accuracy of the simulations by comparison with experimental data, we can begin to examine the molecular level detail provided by the simulations.

The protegrins are a family of five potent cationic antimicrobial peptides originally purified from porcine leukocytes [7,8]. Protegrin (PG)-1 [RGGRL CYCRR RFCVC VGR-amide] has a β-hairpin structure that is stabilized by disulfide bonds linking Cys-6 to Cys-15, and Cys-8 to Cys-13. The broad antimicrobial spectrum of PG-1 includes Gram-positive and Gram-negative bacteria and certain fungi [7,9]; however, PG-1 also damages human cells [10], limiting its therapeutic potential. Hence, though the potent antimicrobial properties of full-length PG-1 made it a reasonable starting point for developing a therapeutic AMP [11], its substantial cytotoxicity was problematic. Indeed, the toxicity of Iseganan (IB-367, a protegrin-like peptide [12]) may have contributed to the unsuccessful outcome of clinical trials to evaluate its ability to prevent oral mucositis and ventilator-associated pneumonia [12-14].

The matter of rationally engineering peptides with antimicrobial yet non-toxic character thus remains open. This study examines the differences between the interactions of two peptides with two types of membrane mimics through various experimental techniques and MD simulations to determine whether the simulation methods accurately capture the interactions between peptides and micelles. PC-72 is a truncated, 11-residue version of PG-1 [LCYCR RRF CV C-amide] and PC-73 is a 10-residue peptide that is identical to PC-72, except for the absence of its N-terminal leucine. The disulfide bond patterns in PC-72 and PC-73 are identical to those in PG-1.

Ideally in the study of AMPs, the interactions of multiple peptides with a lipid bilayer of similar composition to the human or bacterial cell membrane would be examined. Since bilayers pose difficulties with traditional experimental techniques like FTIR and NMR, and due to significant methodological bottlenecks for simulations of peptides with lipid bilayers, we propose to work with micelles as membrane mimics. Micelles provide a minimalistic system for the study of activity and toxicity; like lipid bilayers, micelles possess a well-defined hydrophobic core and a flexible, hydrophilic interface and are commonly used in place of monolayers or bilayers in experimental methods such as NMR spectroscopy [15-18]. Recently, studies of a variety of AMPs including, piscidin, magainin, and aurein, have been conducted in micelles [19-24]. Importantly, they have faster time scales of motion [25-30] and smaller system size, which reduce the required simulation length to one that is computationally feasible. DPC micelles simulate eukaryotic cell membranes, which are generally rich in zwitterionic phospholipids. SDS mimics the negatively charged molecules found in bacterial membranes [31], because it has a flexible, anionic exterior and a hydrophobic interior [32-36]. We have previously studied other AMPs, including protegrin-1 and indolicidin, in SDS and DPC micelles [11,37-42].

We should stress that micelles are not appropriate vehicles for clarifying all the relevant phenomena involved in biological function and pharmacological profiles. Nonetheless, we believe it is a reasonable hypothesis to relate the activity and toxicity of peptides to binding with bacterial and mammalian membrane mimics. One cannot overlook the fact that the peptides need to first bind to the membrane. This is step number one in a cascade of steps that is not entirely clear and may indeed involve the aggregation of multiple peptides to form pore-like structures. What we can try to determine is whether this initial binding is important and to what extent. The present study provides a basis for the use of our simulation methods, which will allow us to continue to investigate this matter.

## 2. Results and discussion

### Depth of insertion

Deuterium exchange experiments were carried out to provide information on the solvent accessibility of the peptides in the two micellar environments. Peptide residues exposed to solvent will exchange very rapidly compared to residues that are buried in more hydrophobic domains of the peptide-micelle ensemble. Both PC-72 and PC-73 deuterated rapidly in the first few minutes of exposure to the solvent. There were no significant differences between the two peptides with regard to the rapid deuteration in either of the micellar systems used in this study. The ready exchange of the micelle-bound PC-72 and PC-73 peptides is more consistent with a position on the solvent-accessible surface of the micelles, rather than enclosure within the hydrophobic interior of the micellar ensemble.

The fluorescence emission of tyrosine was also used to assess the peptides' molecular topography in the micellar systems of SDS and DPC. Tyrosine fluorescence of PC-72 and PC-73 in SDS and DPC centered around 305 nm and had a spectrum (data not shown) that is the same as the peptides in PBS solution alone. This finding suggests that when the peptides are bound to DPC or SDS micelles the tyrosine residue in the amino acid sequence is in an aqueous bulk solution accessible environment. As discussed below, this is consistent with the MD simulations results that show this residue is near the micelle-water interface and bulk solvent accessible.

The depth of insertion of the peptides into the micelles was explored using the molecular dynamics simulation data as well. We began by examining the final, equilibrium conformations of the systems. In figure 2 and 3, final images from the simulations are shown. Visual inspection indicates good agreement between the H/D data and the results of the simulations. In both types of micelles the peptides move to the micelle-water interface, though in each system certain residues are seen to insert into the micelle core. In Figure 2, PC-72 is seen to be located near the DPC-aqueous interface, allowing Tyr-3 to interact with the hydrophobic micelle core and the bulk water. Val-10 and Phe-8 also interact with micelle interface. PC-73 appears to hover just at the interface between the water and micelle surface, with the hydrophobic face of the peptide facing towards the micelle, but not embedded in the core.

**Figure 1:**
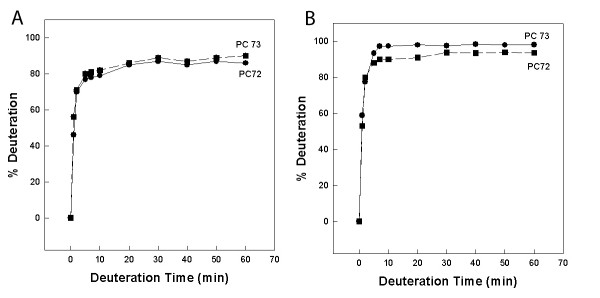
Kinetics of PC-72 and PC-73 deuteration in DPC (A) and SDS (B) micelles.   The percent peptide deuteration was estimated from the area of the amide   II bands as described in Methods

**Figure 2:**
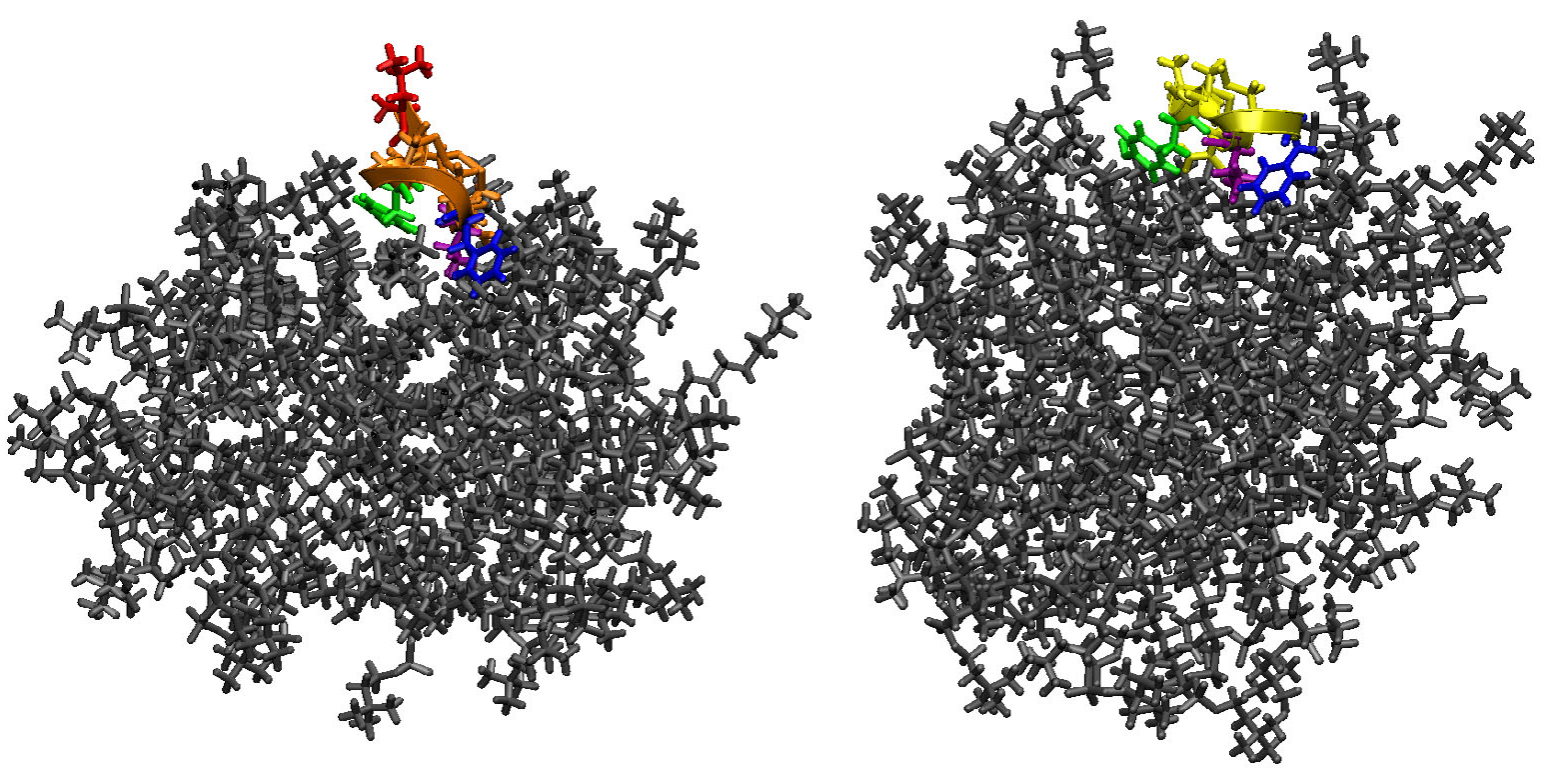
Final views from the simulations in DPC micelles (water removed for clarity). On the left is PC-72, shown in orange, and on the right PC-73, shown in yellow. The hydrophobic residues are colored for emphasis: leucine in red, tyrosine in green, phenylalanine in blue, and valine in violet. In the final conformation for each simulation the peptide is located at the micelle-water interface.

**Figure 3:**
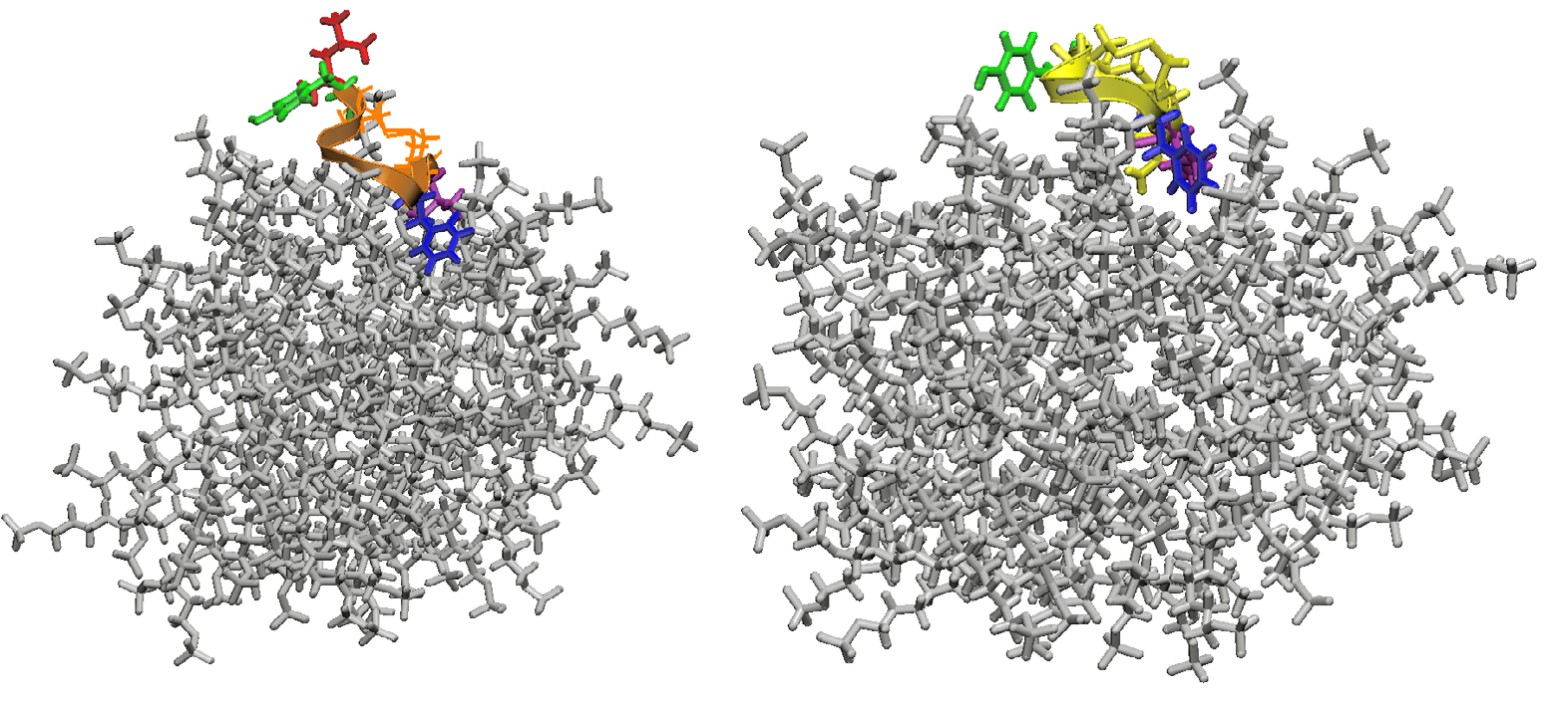
Final views from the simulations in SDS micelles (water removed for clarity). On the left is PC-72, shown in orange, and on the right PC-73, shown in yellow. The hydrophobic residues are colored for emphasis: leucine in red, tyrosine in green, phenylalanine in blue, and valine in violet. Visual inspection suggests that PC-72 is slightly more deeply inserted into the SDS micelle.

In the SDS micelles (Figure 3), PC-72 appears to be tilted at the end of the simulation to allow interactions between Phe-8 and Val-10 and the micelle. In this snapshot from the simulation, Leu-1 and Tyr-3 are not interacting with the micelle. PC-73 shows some insertion of Phe-7 and Val-9 into the SDS micelle core.

**Figure 4:**
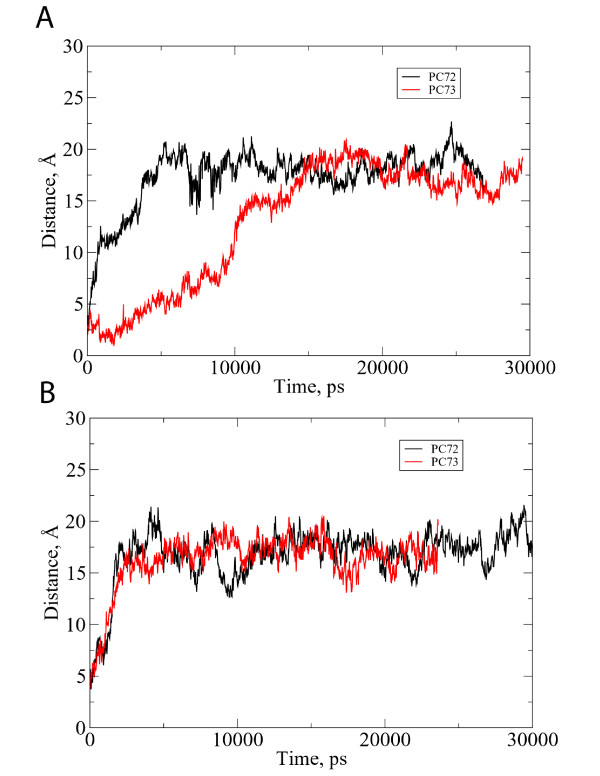
Plots of the distance between the center of mass of the micelle and   center of mass of the peptide.  There is no visible difference for the   peptides in DPC (A) or in SDS (B) micelles, in agreement with the H/D   exchange data.

The distance between the center of mass of the micelle and the center of mass of the peptide was computed for each system, and the results (Figure 4) provide a way to quantify the images in Figures 2 and 3. We also use this measurement as a way to determine when the system has reached equilibrium. In nearly all of the simulations, the peptide reached its equilibrium position with respect to the micelle center of mass within 10 ns, though PC-73 in DPC required nearly 15 ns to equilibrate. There was no discernible difference between the distance from the center of mass of PC-72 and PC-73 in the SDS micelle. Of course, measurements that are based only on the peptide center of mass cannot convey the subtleties of differences in orientation and interaction for these two peptides relative to the micelles.

**Figure 5:**
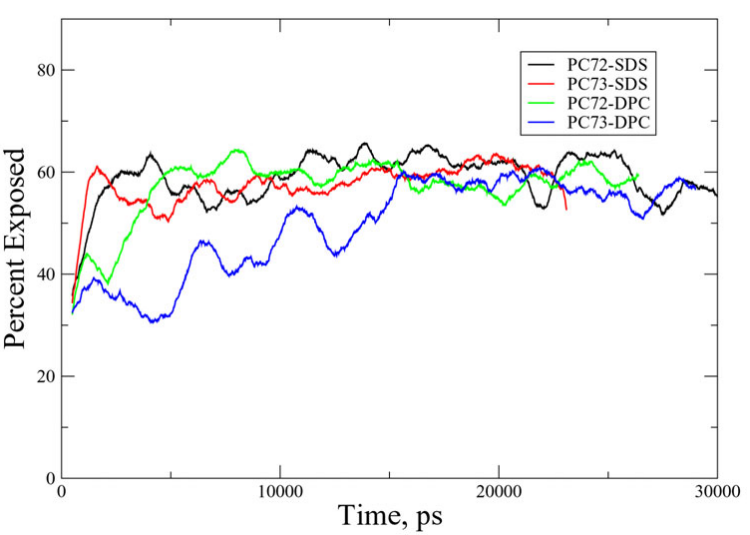
Plot of the percentage of solvent (water) exposed peptide surface area. There are no significant differences between the two peptides in either micelle, in agreement with the H/D exchange data.

The simulations can also provide solvent exposed surface area data for each peptide in each system; we calculated the percentage of the peptide surface exposed to water. The solvent exposed surface area calculated is the Lee-Richards surface area with a probe radius of 1.6 and accuracy set to 0.05 [43]. The results of these calculations are plotted in figure 5 and provide a consistent picture with the H/D exchange data presented earlier: no significant difference between the exposed areas can be detected among the systems.

**Figure 6:**
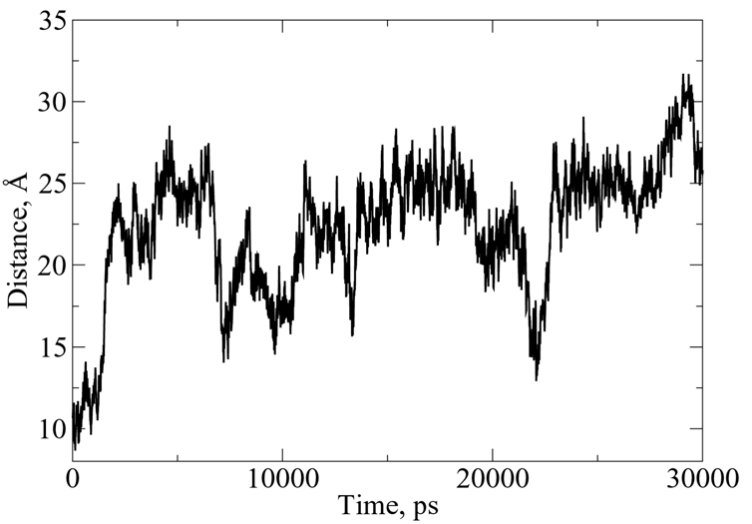
Distance between the leucine residue and the micelle center of mass. It is clear that the position of Leu-1 is fluctuating, moving from its inserted position at 11 ns, and then returning at 20 ns. Because this residue is at the N-terminus, it is not anchored to the interface by proximity to arginine groups like Phe-8.

In order to better understand the subtleties of the position of the individual residues in the micelle, we calculated the distance between the center of mass of the micelle and each residue. For most residues, the relative position of the residue was constant over the period of the simulation in which the peptide has reached its final conformation, with the exception of Leu-1 on PC-72 when interacting with the SDS micelle. In figure 5 we plotted the movement of this residue and show that it moves periodically in and out of the micelle. This is not visible in Figure 2, which shows only the final configuration at 30 ns into the simulation. In Figure 6 we can see that the N-terminus of this peptide interacts intermittently with the micelle core.

**Figure 7:**
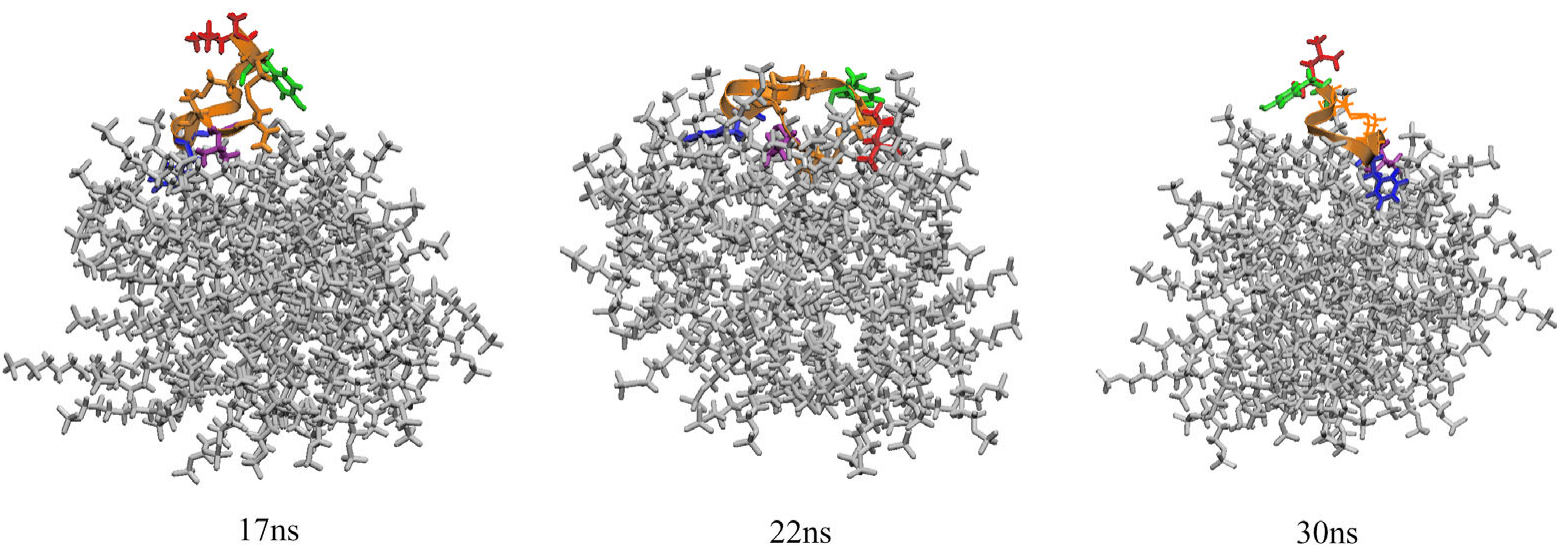
Views of the PC-72 in SDS micelle (water not shown). From the images it is clear that the leucine residue has come out of the micelle and is away from the interface as 17 ns, but attractions to the micelle core pull it back in by 22 ns. Leu-1 moves back out of the micelle by the conclusion of the simulation at 30 ns.

### Secondary structure

Analysis of the dihedral angles from the simulations was used to confirm that a stable, steady-state conformation has been obtained. One would not expect substantial flexibility in the structure of these small, β-hairpin peptides that are constrained by two cysteine-cysteine disulfide bonds, and indeed we see deviations in the value for each dihedral angle to be on the order of 5 to 10 degrees for all of the residues, except for the Ψ angle involving Leu-1 on PC-72 in both SDS and DPC, which exhibit deviations of around 20 degrees. Given that this residue is at the N-terminus, these variations in the angles are not unreasonable.

FTIR measurements of PC-72 and PC-73 peptides were performed in DPC and SDS to provide estimates of the secondary structure of these peptides in each micellar system. Both PC-72 and PC-73 have similar spectral signatures in DPC micelles at a mole ratio of 1:60 peptide to lipid. There are two major dominant absorption peaks at 1674 and 1638 cm^-1 ^that are typical of peptides assuming loop-turn and β-sheet conformations in the micellar environment. The absorbance peaks for β-sheet are broad with a Full Width at Half Maximum (FWHM) of approximately 12 cm^-1 ^indicating that there is a mix of parallel and anti-parallel conformations of the micellar bound peptides. Analysis of the simulation dihedrals for the peptides confirm both the total percent β-sheet as well as the relative contributions of parallel and anti-parallel β-sheet mix of conformations observed in the FTIR measurements (Table 3). The overall percent conformations from β-sheet, loop-turn, helical and disordered conformation also compare reasonably well with those estimated from molecular simulations of both peptides in DPC micelles. PC-72 consistently showed slightly more β-sheet conformers relative to loop-turn structures compared with PC-73 in DPC micelles.

**Figure 8:**
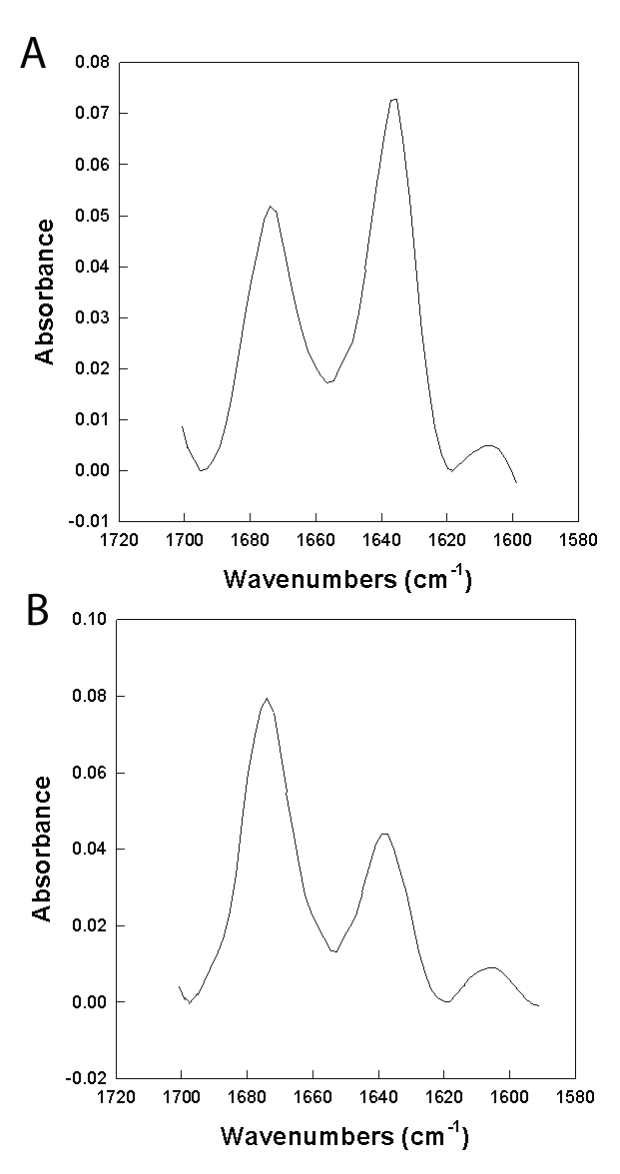
FTIR spectra of PC-72 (A) and PC-73 (B) in DPC micelles.  PC-72 shows   slightly more beta-sheet conformers relative to loop turn structures than   PC-73.

Analysis of PC-72 and PC-73 in SDS micelles showed similar percentages of the various conformations as observed with DPC; however, the β-sheet absorption peak shifted from 1638 cm-1 to 1629 cm^-1 ^and became narrower (FWHM ~8 cm^-1^) suggesting a greater proportion of anti-parallel β-sheet (Figure 8). There was also a clear high frequency band centered around 1689 cm^-1^. This is the signature of frequency splitting of the peptide amide I band into high and low frequency components associated with anti-parallel β-sheets and confirms the presence of a greater population of anti-parallel β-sheet conformations in the SDS micellar environment [44]. Both FTIR measurements and MD simulations suggest that the PC-72 and PC-73 peptides in SDS assume β-sheet anti-parallel conformations to a greater degree than parallel β-sheet. The PC-72 congener has a greater amount of antiparallel β-sheet at the expense of loop-turn conformations than the PC-73 peptide in the SDS micellar environment.

### Strength of interaction

For more detailed information about the interactions between the peptides and micelles, radial distribution functions (RDFs) were calculated from the simulation trajectories. RDFs were calculated for each residue side chain with the SDS and DPC micelle cores. Radial distribution functions tell how likely it is that a certain type of atom will be found in a given distance from another specific atom or atom group, thus giving relative affinities between sets of atoms in the system. A sharper, larger peak indicates stronger interaction.

It can be informative to integrate the area under the plots to determine an overall strength of interaction. Comparing the totals of the integration of all of the residues we find that the sum for PC-72 in SDS (when Leu-1 is inserted into the core) is 54.8, larger than the sum for PC-73, 39.3. In DPC, there is a less significant difference, the peaks total 49.7 for PC-72 and 41.1 for PC-73.

There are several residues that show strong interactions with the SDS micelle core. Leu-1 on PC-72 has a large peak, when calculated during the "inserted" time period, suggesting that it is of importance for this peptide's activity. The neighboring Cys-2 residue also exhibits a strong peak, thereby signifying the importance of the N-terminus in interactions with the negatively charged micelle. There are strong peaks for Phe-8 and Val-10 on with the SDS micelle as well, though they are not much more significant than the peaks from the corresponding residues on PC-73.

In comparing the two peptides interactions with DPC, we see few differences for each residue. There are slightly stronger interactions around Arg-5 on PC-72 and slightly stronger interactions at Phe-7 on PC-73, but the differences offset each other, as evidenced by the integration results.

If we contrast the interactions of PC-72 with both types of micelles, we see a clear difference in the interactions of Tyr-3 and, most notably, a distinct lack of peak for Leu-1 on PC-72 with DPC. The RDF for Leu-1 on PC72 fluctuates, and is shown for the period around 22 ns (the higher peak) and for the last 5 ns of the simulation (the lower peak). Tyr-3 has little interaction with the SDS micelle core, but has a much stronger peak with the DPC micelle core. This appears to be at odds with the fluorescence data discussed previously; however, it can be explained by the images in figure 2 and 3, which show how Tyr-3 is interacting with the micelle core, but is also exposed to the water.

## 3. Conclusion

We have presented the results of detailed investigations into four systems: two related peptides in SDS and DPC micelles, using a combination of experimental techniques and molecular dynamics simulations. We see that the simulation results complement the experimental results. In comparing the FTIR and the simulation results, we see that the peptides are adopting similar conformations in the experimental setting as in the simulations. This assuages some of the concerns about the ability of the relatively short time scale of the simulations to capture the proper peptide structures. Additionally, from the deuterium exchange data, it appears that the peptides all position themselves at the surface of the micelle, where they can interact with both the micelle core and the bulk water. From the fluorescence data, it seems that Tyr-3 on PC-72 (and Tyr-2 on PC-73) is able to interact with the bulk water when interacting with both types of micelle. Though the radial distribution function for Tyr-3 on PC-72 when interacting with DPC has a high peak, we can inspect the system visually and see that though Tyr-3 is in contact with the micelle core, it is also exposed to the water subphase.

Of particular note in this study is the observed difference in the interactions of the N-termini of PC-72 and PC-73 with the SDS micelle in the simulation, differences that could not be observed from experiments. Though there is little difference in the experimental data between the two peptides when interacting with the SDS micelles, we do find that the leucine residue on PC-72 is interacting with the micelle core, albeit intermittently.

The agreement between the experimental and simulation data demonstrates the validity of the simulation methods for investigating the interaction between peptides and micelles. Additionally, the simulations provide the necessary level of detail to determine differences in the ways in which the peptides interact with the two types of micelles and knowing that the simulation results agree with experimental results, we can then extend the analysis of the simulations to explore the systems in more detail. This work is a necessary step in the overall goal of developing simulation methods to determine activity and toxicity of peptides a priori, though the methods must be developed through the study of more peptides. It would be premature to base conclusions about the effectiveness of the micelles as membrane environments based on two data points, PC-72 and PC-73.

## 4. Methods

### 4.1 Peptide synthesis and purification

PC-72 and PC-73 were synthesized on a 0.25 mmole scale with an Applied Biosystems 431A peptide synthesizer using FastMoc™ chemistry [45], double coupling, and Rink amide MBHA resin (Novabiochem, San Diego, CA). The cleaved peptide was deprotected with a solution of trifluoroacetic acid:ethanediothiol:thioanisole:water,10:0.25:0.5:0.5, v:v for 2 hours, followed by precipitation with cold t-butyl ether. After drying under vacuum, the precipitate was reduced with Tris(2-carboxyethyl)phosphine hydrochloride (TCEP, Pierce, Rockford, IL), and purified by reverse phase HPLC on a C18 column (Vydac, Hesperia, CA) using a linear gradient of water:acetonitrile with 0.1% TFA as an ion pairing agent. Folding (air oxidation) of the purified, reduced peptide (0.1 mg peptide/ml buffer) was done in 5 mM ammonium acetate buffer pH 7.5 for 48 hours at 25°C with stirring. The oxidized peptide was purified by the above HPLC procedure and its mass was confirmed by MALDI MS. Peptides were twice freeze-dried from 10 mM HCl to remove residual trifluoroacetate counter ions that would interfere with FTIR measurements.

### 4.2 Fluorescence measurements of tyrosine fluorescence in micelles

Steady-state fluorescence emission spectra of tyrosine residues in PC-72 and PC-73 were made at 25°C, in DPC and SDS micelles, with a Cary Eclipse Fluorescence spectrophotometer at an excitation wavelength of 274 nm. The molar ratio of peptide to SDS (Sigma, St. Louis, MO) or DPC (Avanti Polar Lipids, Alabaster, AL) was ~1:60. Reagents were prepared in a buffered saline solution (8.1 g/L NaCl with 0.6 g/L K_2_HPO_4_, pH 7.5; Mediatech, Herndon, VA) that closely matched the simulation conditions.

### 4.3 FTIR measurements of peptide conformation and hydrogen-deuterium exchange

Infrared spectra were recorded at 25°C using a Vector 22 FTIR spectrometer (Bruker Optics, Ettingen, Germany) equipped with Deuterated Triglycine Sulfate (DTGS) detector, averaged over 256 scans at a gain of 4 and a resolution 2 cm^-1^. To obtain Fourier-transform infrared (FTIR) spectra of PC-72 and PC-73 peptides in SDS or DPC micelles, the micelle preparations (peptide:SDS/DPC molar ratio ~1:60) were allowed to form a film by air drying the aqueous dispersion on 50 × 20 × 2 mm 45° attenuated total reflectance (ATR) crystals fitted for the Vector 22 spectrometer (Pike Technologies, Madison, WI, USA). The sample was then hydrated by passing deuterium vapor in nitrogen gas over the film one hour prior to measurement.

The amide I bands of FTIR spectra of the PC-72 and PC-73 peptides were analyzed for various secondary conformations [27]. The proportions of α-helix, β-turn, β-sheet, and disordered conformations were determined by Fourier self-deconvolutions for band narrowing and area calculations of component peaks determined with curve-fitting software supplied by Galactic Software. The frequency limits for the different structures were as follows: α-helix (1662 to 1645 cm^-1^), β-sheet (1643 to 1623 and 1695 to 1685 cm^-1^), β-turns (1682 to 1662 cm^-1^) and disordered or random (1650 to 1637 cm^-1^) [46]. Although anti-parallel beta sheets have low frequency amide I band centered around 1630 cm^-1 ^and a less intense high frequency signature band in the range of 1685 to 1695 cm^-1^, parallel beta sheet amide I bands are less definitive [37]. Recent studies of proteins with a large proportion of parallel beta strands suggest that the amide I absorption for this conformation is centered about 1638 cm^-1 ^and therefore can overlap contributions from the anti-parallel amide I absorbance [47] making spectral deconvolution of these two conformations problematic. For this reason we report the overall integration of the spectral region from 1643 to 1623 cm^-1 ^as β-sheet conformations that includes both parallel and anti-parallel conformations.

The time course of deuterium exchange was determined by subjecting the peptide-micelle films in the sealed ATR sample chamber described above to a stream of D2O-saturated nitrogen gas. The course of sample deuteration was monitored by acquiring FTIR spectra at various time points over the period of one hour. The relative area of the amide II band between 1596 and 1502 cm-1 was used as an index of the degree of sample deuteration.

### 4.4 Molecular dynamics simulations

Simulations of PC-72 and PC-73 in SDS and DPC micelles were carried out as previously described [48,49]. Structures for PC-72 and PC-73 were created by homology modeling as described in [50]. Briefly, the known structure for PG-1 was imported into MOE [51] and the N- and C-termini were removed and the structure minimized using the AMBER89 forcefield. Once imported into CHARMM, the C-terminus is amidated. Because these two peptides are small (10 and 11 residues), and their sequences are identical with the corresponding protegrin-1 regions, and they are constrained by the same two disulfide bonds, we can be confident that the structures from homology modeling with a protegrin-1 template are very close to the actual structures; that is, that they adopt the cysteine-cysteine constrained β-hairpin structure.

The starting coordinates of the SDS micelle-water complex were taken from simulations carried out by MacKerell [52]. The SDS micelle was composed of 60 molecules and solvated in a cube with 54.15Å long sides that contained 4375 water molecules. In previous simulations of protegrins with the DPC micelle, we saw the separation of one molecule of DPC from the micelle, suggesting a lower N_aggregation _than in the original 60 molecule DPC micelle. To correct for this, one molecule was removed from the micelle resulting in a micelle composed of 59 DPC molecules. Due to the slightly larger size of a DPC molecule versus SDS, the DPC micelle was solvated in a rhombic dodecahedron containing 6120 water molecules. In creating a larger simulation box, the goal was to increase the distance between the system and the edges of the box without increasing the number of water molecules necessary to solvate the box unnecessarily. The rhombic dodecahedron geometry allows a thicker layer of water around the micelle while increasing the actual number of atoms that must be simulated within a reasonable amount. In both cases, the cell dimensions were set to obtain the equilibrium bulk water density away from the micelle interface of 0.033/Å ^3 ^and as we have seen, the surfactant molecules are able to rearrange themselves as necessary to accommodate changes in the aggregation number due to the presence of the peptide. The choice of using a preformed micelle with a set aggregation number is justified by scores of simulations of peptides in micellar systems conducted, obtaining microseconds of trajectories in total [11, 37, 38, 39, 40, 41, 42, 48, 49, 60]. During all these simulations we have only observed the departure of a single DPC molecule from a micelle composed of 60 molecules. If the systems were not at equilibrium, even rare occurrences of destabilization would have been observed more than once. We thus feel confident that our system is with the range of aggregation numbers for DPC-peptide and SDS-peptide systems. Water was modeled using the TIP3P potential [53]. NaCl ions at a concentration of 0.15 M were randomly distributed in the aqueous phase for the SDS and the DPC simulations.

In all simulations, the peptide was initially placed in the center of the micelle core with the micelle center of mass overlapping the peptide's center of mass. Given the spherical symmetry of the micelle, the orientation of the peptide is unimportant. The system is inspected visually to ensure that no serious overlaps occur. Then, to further remove initial bad contacts between the peptide and the core, and to prevent penetration of water during equilibration, the system was minimized with the peptide and bulk water initially kept under weak harmonic constraints with spring constants of 10 and 5 kcal/mol Å, respectively. The constraints were gradually removed in 20,000 steps of minimization, using the steepest descent method. The entire system was then minimized for 20,000 additional steps, without constraints. Thereafter, the system, consisting of approximately 16,000 atoms, was gradually heated to 303.15 K. After 500 ps of equilibration, the entire assembly was subjected to NPT dynamics at a pressure of 1 atm and a temperature of 303.15 K. The constant pressure-temperature module of CHARMM was used for the simulations with a leap-frog integrator (2 fs time step). The temperature was set at 303.15 K using the Hoover temperature control [54]. All the components of the piston mass array were set to 500amu for the extended system pressure algorithm [55,56]. The electrostatic interactions were simulated using the particle mesh Ewald (PME) summation[57] without truncation and a real space Gaussian width of 0.25 Å ^-1^, a β-spline order of 4, and a FFT grid of about one point per Angstrom. All simulations were carried out using CHARMM version c30b2 with the param22 parameter set [58]. The CHARMM program, and its force field and parameters are described in detail by both Brooks [58] and MacKerell et al [59]. Simulations were stopped approximately 10 nanoseconds after the peptide showed no change in its location from the center of the micelle.

## 5. Abbreviations

MD, molecular dynamics; AMPs, antimicrobial peptides; CHARMM, Chemistry at Harvard Macromolecular Mechanics; PG-1, protegrin-1; SDS, sodium dodecylsulfate; DPC, dodecylphosphocholine, FWHM, full width at half maximum of the FTIR spectral band

## 6. Authors' contributions

AJW carried out the FTIR, H/D exchange, and fluorescence experiments. AAL carried out the molecular dynamics simulations. YNK conceived of the study and participated in its design and coordination and helped draft the manuscript. All authors read and approved the final manuscript.

**Figure 9:**
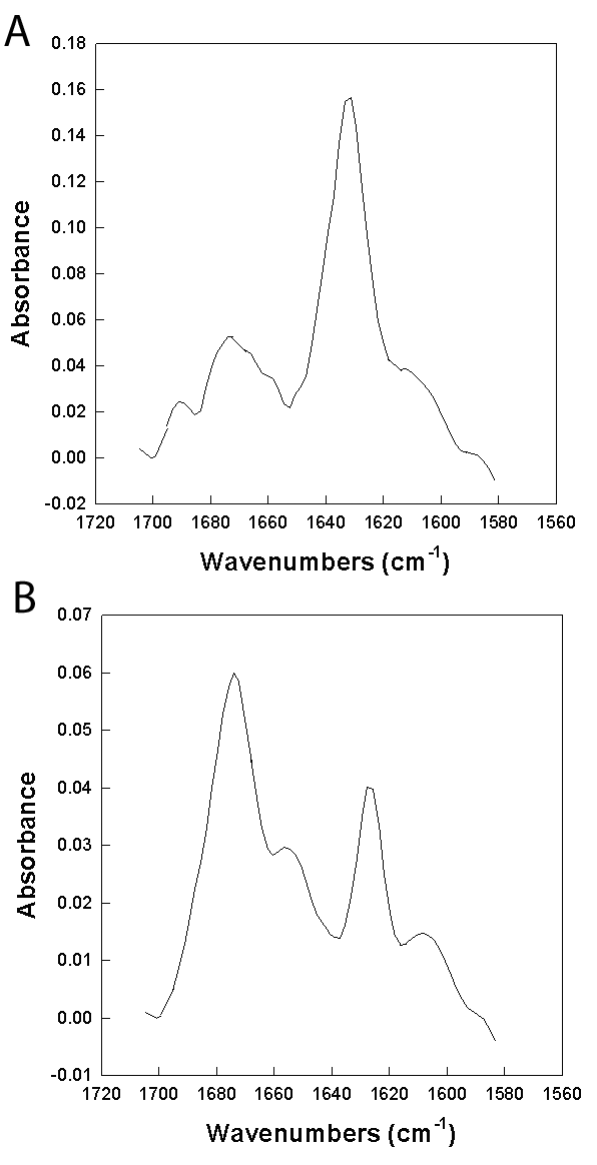
FTIR spectra of PC-72 (A) and PC-73 (B) in SDS micelles.

**Figure 10:**
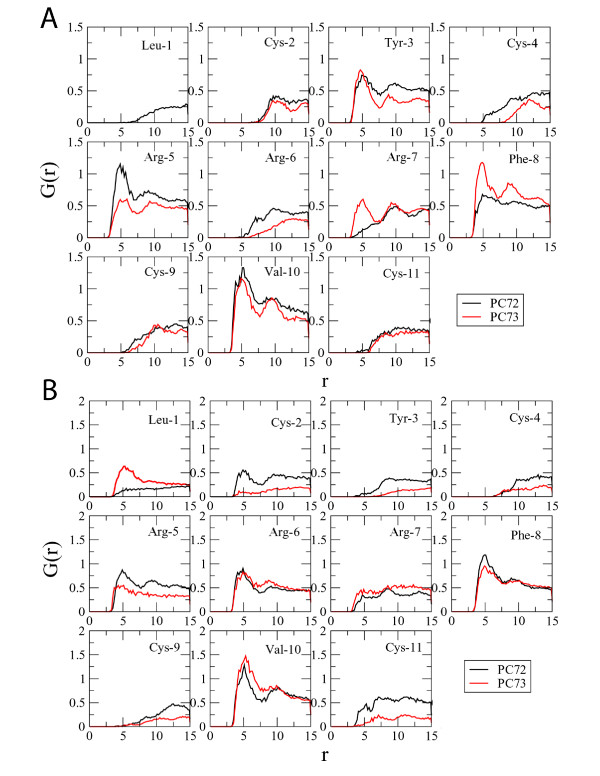
Radial distribution functions for PC-72 (A) and PC-73 (B) with the   micelle cores.  There are few differences between the RDFs for PC-72 and   PC-73 in DPC.  In SDS, there is a strong peak for Leu-1 for the 20ns to   25ns time period (in green), suggesting that this residue is responsible   for the activity of this peptide, though it is not constantly inserted   into the micelle.

**Table 1:** Average dihedral angles for PC-72 over the last 5 ns of simulation with deviation in parentheses

	**DPC**		**SDS**	
	**Φ**	**Ψ**	**Φ**	**Ψ**
**1**		-176.3 (25.0)		144.9 (18.6)
**2**	-97.9 (8.5)	159.7 (12.2)	-82.3 (8.2)	130.5 (10.3)
**3**	-102.0 (7.1)	93.7 (7.1)	-125.0 (11.9)	165.5 (6.2)
**4**	-102.3 (4.9)	148.2 (11.6)	-108.3 (9.6)	136.5 (8.29)
**5**	-137.2 (11.4)	-79.0 (5.9)	-93.1 (6.2)	-79.6 (9.2)
**6**	-109.1 (6.9)	-84.0 (5.9)	-103.6 (8.0)	-94.9 (7.9)
**7**	-103.8 (6.9)	-66.1 (4.6)	-99.7 (8.0)	-60.3 (4.5)
**8**	-84.0 (5.0)	133.7 (8.3)	-86.6 (5.0)	109.8 (7.0)
**9**	-114.2 (9.3)	134.7 (8.3)	-92.1 (5.3)	73.3 (9.0)
**10**	-104.9 (7.0)	121.1 (9.3)	-102.5 (9.1)	133.8 (8.5)
**11**	-105.5 (8.0)		-105.6 (10.0)	

**Table 2:** Average dihedral angles for PC-73 over the last 5 ns of simulation with deviation in parentheses

	**DPC**		**SDS**	
	**Φ**	**Ψ**	**Φ**	**Ψ**
**1**	0	167.9 (7.8)	0	155.0 (13.4)
**2**	-97.9 (7.0)	93.7 (5.1)	-115.5 (15.6)	136.5 (10.8)
**3**	-97.5 (4.7)	135.4 (6.5)	-105.3 (14.7)	131.4 (8.8)
**4**	-109.0 (5.8)	-144.2 (9.7)	-96.2 (8.7)	-74.1 (7.7)
**5**	-83.1 (9.1)	-56.1 (5.1)	-102.0 (7.6)	-99.5 (10.3)
**6**	-86.8 (6.1)	-57.8 (6.7)	-102.3 (9.6)	-60.0 (4.7)
**7**	-105.7 (7.6)	164.4 (5.0)	-88.1 (5.7)	121.6 (8.7)
**8**	-135.7 (5.4)	132.2 (5.3)	-94.6 (6.7)	69.2 (9.6)
**9**	-109.3 (6.3)	120.7 (5.8)	-91.6 (8.2)	130.7 (8.8)
**10**	-100.8 (5.7)	0	-116.1 (12.5)	0

**Table 3:** Proportions of secondary structure in micelles for PC-72 and PC-73 from FTIR spectra and molecular dynamics simulations*. Data are the mean of 5 separate determinations.

Sample	% Beta sheet	%Loop-turn	%Disordered	%Helix
PC-72 SDS FTIR	43.3	42.3	4.4	10.0
PC-72 SDS Simulation	52.0	27.2	14.4	6.4
PC-72 DPC FTIR	54.0	30.8	10.2	5.0
PC-72 DPC Simulation	54.2	27.4	9.3	9.1
PC-73 SDS FTIR	23.4	48.1	18.5	10.0
PC-73 SDS Simulation	40.0	29.0	20.0	11.0
PC-73 DPC FTIR	38.6	46.1	6.3	9.0
PC-73 DPC Simulation	43.0	27.0	15.0	15.0

*The percentage residue specific anti-parallel and parallel beta sheet conformations were determined from peptide simulation structures using the Hyperchem 7.5 secondary structure analysis utility. This utility classifies anti-parallel beta sheet as residues having dihedral angles of Φ = -139° and ψ = 135° and parallel beta sheet as residues having dihedral angles of Φ = -119° and ψ = 113°. The values in the above table represent the sum of residues participating in anti-parallel and parallel beta sheet structures for a given peptide in a specific environment. Similar analysis procedures were used for helix and random conformations. Loop structure percentages were based on the residues participating in disulfide stabilized loop sequence since there is no specific secondary structure classification for this motif in the analysis utility.
